# Geometric stoichiometry model yields relevant insights for assessing nutrient-related environmental impacts of aquaculture

**DOI:** 10.1093/conphys/coaf066

**Published:** 2025-09-15

**Authors:** Sowdamini Sesha Prasad, Duncan D Cameron, Chris G Carter, Andrea Williamson, Julia L Blanchard

**Affiliations:** Institute for Marine and Antarctic Studies (IMAS), University of Tasmania Private Bag 129, Hobart, Tasmania 7001, Australia; Department of Earth and Environmental Sciences and Manchester Institute of Biotechnology, The University of Manchester, John Garside Building, 131 Princess Street, Manchester M1 7DN, United Kingdom; Institute for Marine and Antarctic Studies (IMAS), University of Tasmania Private Bag 129, Hobart, Tasmania 7001, Australia; Institute for Marine and Antarctic Studies (IMAS), University of Tasmania Private Bag 129, Hobart, Tasmania 7001, Australia; Institute for Marine and Antarctic Studies (IMAS), University of Tasmania Private Bag 129, Hobart, Tasmania 7001, Australia; Centre for Marine Socioecology, University of Tasmania, Hobart, Tamania 7001, Australia

**Keywords:** Aquaculture, aquaculture models, ecosystem models, eutrophication, food security, global food change

## Abstract

Aquaculture plays a crucial role in global food security and is being increasingly used to aid species and ecosystem conservation. However, concerns over environmental impact of aquaculture expansion are driving research into ecosystem approaches to aquaculture. Ecosystem approaches to aquaculture require understanding of the relationship between aquafeeds and aquaculture species to maximize consumer growth, quantify elemental flow of nutrients and minimize waste output. Conventional bioenergetic models typically assume fixed elemental ratios to quantify metabolic processes and do not consider an organism’s nutrient demand. A new bridging framework, Geometric Stoichiometry (GS), unifies nutritional geometry and ecological stoichiometry disciplines using macromolecules as currencies and dietary regulation to balance nutrient deficits and excesses by the consumer. We present the first application of the GS framework to aquaculture by investigating how different formulated feed ingredients affect intakes to maintain C:N homeostasis, growth and waste output using three opportunistic datasets for an emerging aquaculture species, slipper lobster (*Thenus australiensis*). Our GS model results indicate that protein sources and their inclusion levels drive the most variation in feed intake and growth. It also predicts highest nitrogenous waste for fish meal and lowest for squid by-product meal feeds. Our results highlight the need for targeted experiments to further refine the GS model to help support environmental management and formulate low-impact feeds for aquaculture.

## Introduction

Aquaculture is the fastest growing global food sector and plays a crucial role in global food security (Froehlich *et al.,* 2018). However, there are concerns about the environmental consequences of aquaculture expansion, primarily due to waste production and its release into the environment. There is considerable evidence that aquaculture waste from uneaten feeds and faeces can lead to an increase in primary productivity, resulting in eutrophication and dead zones in freshwater and marine systems (Fry *et al.,* 2016). Progress in aquaculture feed design and nutrition has aimed to minimize waste and maximize absorption and assimilation of nutrients (Carter and Codabaccus, 2022; Elvines *et al.,* 2024), including shifting from fresh feeds to formulated, pelleted feeds. Despite these advancements, aquaculture waste continues to pose a significant environmental challenge. While the term “aquaculture” is typically associated with species reared for human consumption, it is also becoming an increasingly popular and important tool for enhancing fisheries stock abundance (Lorenzen, 2005; Lorenzen *et al.,* 2021) and conservation of threatened and vulnerable aquatic species (Froehlich *et al.,* 2017). As the importance of aquaculture grows, from feeding humans to species and ecosystem conservation, it is crucial to understand and accurately estimate waste impacts associated with different aquaculture feeds and species.

Numerous studies have shown that the type and quality of aquaculture feeds influence the chemical composition (stoichiometry) of the resulting waste (Wang *et al.,* 2013; Kokou and Fountoulaki, 2018), which highlights the need for ongoing research and development of optimal feeds that enhance nutrient assimilation and reduce waste. Typically, aquafeeds are formulated to meet the physiological and nutritional demands of organisms throughout life stages (Jobling, 2015; Hardy *et al.,* 2022), and a wide range of models are used to assess the efficiency of feeds and feed ingredients (Chary *et al.,* 2022). For instance, traditional fish bioenergetic models, typically parameterized using aquaculture digestibility and growth experiments, assume that all energy is derived from the feed, a fraction is lost via egestion and the remainder is assimilated and allocated to different metabolic processes: respiration, maintenance, growth and nitrogenous excretion (Chary *et al.,* 2022; Kaushik and Schrama, 2022; Carter *et al.,* 2025). Conventional bioenergetic models generally assume that nutrient ratios would be maintained from feed to fish to waste and do not necessarily track different nutrient components and their ratios separately. Additionally, they do not consider the organism’s nutrient demand into account and assume that they feed impartially. Virtual technologies and models have been used to assess and predict environmental impacts of aquaculture and allow ecosystem approach to aquaculture (EAA) principles to be translated into practice (Kokou and Fountoulaki, 2018; Ferreira *et al.,* 2021). Nevertheless, we still lack an integrative understanding of how different nutrient ratios in feeds propagate through organisms and into the environment, which is essential for accurately estimating environmental impacts (Wang *et al.,* 2012).

There exists a large body of theory surrounding how organisms’ nutritional demand influences food capture and utilization, including ecological stoichiometry (ES) (Sterner and Elser, 2009; Schiettekatte *et al.,* 2020) and nutritional geometry (NG) (Raubenheimer *et al.,* 2009). Both approaches emphasize organism nutrition and homeostasis but differ in their origin and goals. NG, originating from behavioural ecology, explores interactions between nutritional and non-nutritional components of an organism’s nutrition to determine which food components influence food selection, physiological regulation and their associated fitness outcomes (Sperfeld *et al.,* 2016). It thereby explains animals’ feeding behaviour and physiology with reference to food components and their interactions (Simpson and Raubenheimer, 1995; Raubenheimer *et al.,* 2009; Raubenheimer and Simpson, 2018). Ecological stoichiometry, originating from biogeochemistry (Sperfeld *et al.,* 2016), uses elements and their ratios to relate to macronutrients or other biochemical components of organisms and ecosystems. Ecological stoichiometry assumes that nitrogen (N) is located primarily in proteins, phosphorus (P) is mainly present in bones of vertebrates (Sterner and Elser, 2002, 2009) and carbon (C), although a structural component of all other molecules, is mainly present in lipids and carbohydrates (Elser *et al.,* 1996). NG has been used in aquaculture to identify optimal diet composition (Ruohonen *et al.,* 2007), which should also reduce animal waste and ecological outcomes. However, the NG framework on its own does not track elements beyond intake, which is required for quantitative understanding of how organismal demand of macromolecules (e.g. proteins, lipids, carbohydrates) could affect nutrient waste in the environment (e.g. nitrogenous waste).

**Figure 1:**
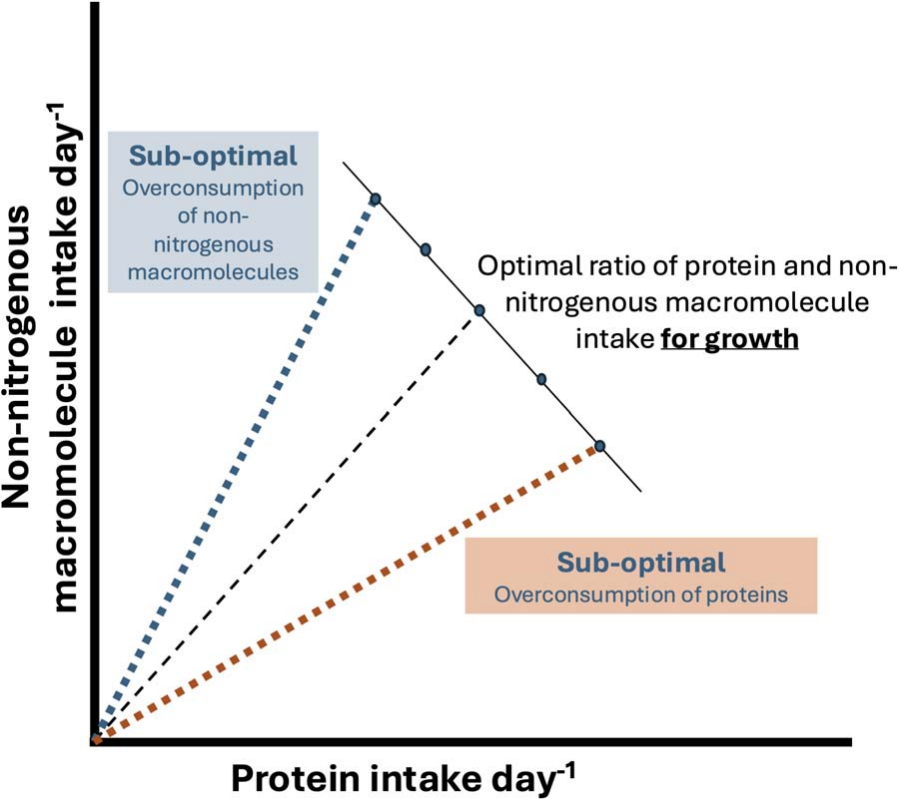
A simple diagram showing intakes of protein and non-nitrogenous macromolecule (carbohydrates or lipids) for a hypothetical organism (this could be a single-celled bacteria or humans). Different rails (orange and blue dotted lines) represent different feeds with their own protein/non-nitrogenous macromolecule ratio. For every organism, there is an optimal protein/non-nitrogenous macromolecule ratio (black dashed line) where growth is maximized and anything over or under is sub-optimal (adapted from Anderson *et al.,* 2020).

A recent advancement, Geometric Stoichiometry (GS) (Anderson *et al.,* 2020), fills this gap by quantifying nutrient ratios as they move from feed through organisms and into waste. It builds on the equations of ecological stoichiometry and incorporates rules of compromise associated with nutritionally imbalanced foods to generate state-space diagrams like NG (Fig. 1). The framework uses macromolecules (proteins, lipids and carbohydrates) as currencies and calculates intakes required to give rise to a specific growth rate by accounting for energetic costs associated with feeding, absorption, assimilation, maintenance and growth (new biomass). It then quantifies carbon and nitrogen budgets required to calculate respiration, excretion and egestion based on the C:N ratio of feeds, the organism (representing homeostasis) and the metabolic and maintenance costs. GS also includes a metabolic penalty to mechanistically explain reduced growth when protein is provided in excess, i.e. when an organism is fed nutritionally imbalanced diets (Anderson *et al.,* 2020). The metabolic penalty is especially relevant for aquaculture to optimize feed design from nutrition perspective (balanced diets) and minimize waste as excess proteins translate into nitrogen waste into the environment (Ruohonen and Kettunen, 2004; Boyd *et al.,* 2020; Glencross, 2020; Cooney *et al.,* 2021; Tacon *et al.,* 2022). While GS was originally parameterized for zooplankton, our study is the first to explore its potential applicability to an aquaculture species: *Thenus australiensis* (Codabaccus *et al.,* 2020; Landman *et al.,* 2020; Wirtz *et al.,* 2022a), commonly known as slipper lobster or Moreton Bay bug. Lobsters are an ideal case study because they are an important and highly valuable developing aquaculture species with high demand and limited supply (Jeffs, 2010). There have been many nutritional studies to ascertain viability of formulating feeds to replace fresh feeds, which are known to create more waste (Perera and Simon, 2015).

In this study, first, we demonstrate how data typically collected in aquaculture experiments could be used to parameterize and calibrate GS models. Then, using the GS model calibrated for three published experimental datasets, we explore two questions:

(i) How do different inclusion levels of feed ingredients affect crude protein (CP) and total lipid intakes required to maintain C:N homeostasis and growth? and (ii) How do different CP percentages in feeds (C:N) influence the nitrogenous (N) waste output?

## Materials and Methods

### Model parameterization with experimental data

**Figure 2:**
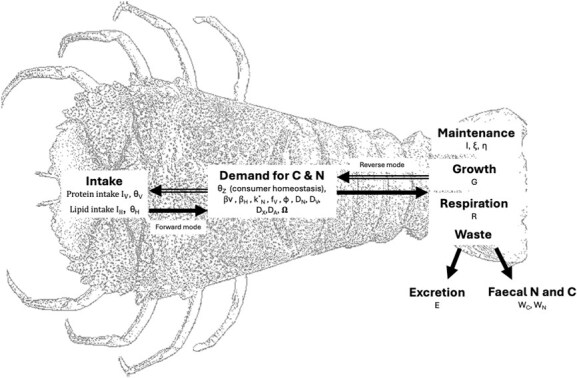
GS model applied to slipper lobster. The model describes how macromolecule intake is regulated by the organism’s demand for macronutrients to maintain homeostasis (represented by C:N ratio of consumer, θz) and to support growth (G) using carbon (C) and nitrogen (N) budgets. The model can be applied in two ways: (1) in reverse mode, where a known growth rate is used to predict the required intake and the resulting C and N budgets for metabolic processes and waste production; or (2) in forward mode, where known intake values are used to estimate growth, C and N allocation and waste outputs.

The GS model equations (Table S1) can operate in two modes: forward and reverse mode. Forward mode starts from intake of each macromolecule to estimate growth, respiration and waste, after accounting for demand and utilization (Fig. 2). However, detailed intake rate data normalized to consumer biomass required to run the model in forward mode are often not known. Reverse mode starts instead with a given growth rate, and a defined fraction of protein for energetic costs (fV), to back-calculate required combinations of protein and non-nitrogenous macromolecule (lipid or carbohydrate) intakes that are needed to give rise to that growth rate. Detailed parameter definitions and values are provided in supplementary information (Table S2). For a more detailed model description, see Anderson *et al.* (2020). To apply the GS model (Anderson *et al.,* 2020) to *T. australiensis* (hereafter referred to as slipper lobster), we required information on feed composition, feed intake, feed ingredient digestibility (also referred to as absorption), feed-specific growth rates and C:N ratio of the organism as model inputs. We also required C:N ratio of proteins, specific dynamic action (SDA), biomass turnover, other basal costs, nitrogen synthesis efficiency (hereafter referred to as protein synthesis efficiency as animals cannot synthesize nitrogen) and penalty function as input model parameters (parameter definitions and data sources are detailed in the supplementary information (Table S2).

We selected three opportunistically available datasets for slipper lobster, a species of commercial aquaculture interest for applying the geometric stoichiometric framework in aquaculture. We provide a brief overview of the experimental methodology here.

All three studies investigated the influence of different feed ingredients on the growth performance of slipper lobster. The experiments ran for 9–12 weeks, and the feeds were formulated to contain different protein sources (Experiment 1: Wirtz *et al.,* 2022a, 2022b), inclusion levels of phospholipids (Experiment 2: Landman *et al.*, 2021a) and inclusion levels of fresh feeds in formulated feeds (Experiment 3: Landman *et al.*, 2021b) (Table 1). Experimental trials were conducted on J3–J7 juvenile lobsters with a stocking density of 65–110 lobsters m^−2^. Because chemical composition of the feeds, lobsters and faeces were all analysed, macromolecule-specific feed intake information, apparent digestibility of feed ingredients, as well as overall tank-level growth rate data were available. These estimates were then converted into units that are required for the GS model (shown in Table 1).

**Table 1:** Experimental data used as inputs for GS model applied to slipper lobsters

Experiment	Feed sources	Protein intake (mol C mol C^−1^ day^−1^)	Protein digestibility	Lipid intake (mol C mol C^−1^ day^−1^)	Lipid digestibility	Growth rate (day^−1^)
1	FM	0.02750	0.796 ± 0.033	0.00833	0.612 ± 0.14	0.0043 ± 0.03
	Krill meal (KM)	0.02472	0.863 ± 0.016	0.00829	0.672 ± 0.018	0.0051 ± 0.03
	Soybean meal (SBM)	0.02806	0.913 ± 0.015	0.00880	0.503 ± 0.077	0.0057 ± 0.03
	Squid by-product meal (SWM)	0.03392	0.949 ± 0.015	0.01515	0.665 ± 0.054	0.006 ± 0.09
2	D1	0.02766	–	0.00426	0.86 ± 0.0118	0.0126 ± 0.09
	D2	0.02552	–	0.00428	0.823 ± 0.016	0.0109 ± 0.06
	D3	0.02418	–	0.00411	0.814 ± 0.032	0.011 ± 0.09
	D4	0.02537	–	0.00453	0.769 ± 0.054	0.0102 ± 0.11
	D5	0.02693	–	0.00498	0.797 ± 0.047	0.0123 ± 0.07
	D6	0.02615	–	0.00504	0.805 ± 0.072	0.011 ± 0.07
3	BM0%	0.07381	–	0.02098	–	0.0233 ± 0.10
	BM1.6%	0.07473	–	0.02090	–	0.027 ± 0.05
	BM3.1%	0.07889	–	0.02283	–	0.0243 ± 0.13
	BM6.3%	0.07844	–	0.02179	–	0.023 ± 0.06
	BM12.5%	0.06118	–	0.01658	–	0.0235 ± 0.04
	BM25%	0.06965	–	0.01770	–	0.0252 ± 0.04
	BMHS	0.08270	–	0.01721	–	0.0326 ± 0.07

Fields marked as ‘–’ were not measured by the experiments, in which case, model defaults were used. Experiment 1: Wirtz *et al.* (2022a, 2022b); Experiment 2: Landman *et al.* (2021a); Experiment 3: Landman *et al.* (2021b).

To parameterize the GS model with biomass-specific units (rates normalized to consumer biomass), we applied the following widely accepted conversion factors (Carter *et al.,* 2025).

- Protein: calculated as N ^*^ 6.25
- Lipid: assumed that 75% of a lipid (triglyceride) is carbon
- C:N ratio of protein in feed: assumed to be 3.87 mol C (mol N)^−1^
- Lobster carbon content: estimated that 1 gram of lobster contains 0.41 grams of carbon (Codabaccus *et al.*, unpublished)
- Molar mass conversions for N = 14.007 g mol^−1^ and C = 12.011 g mol^−1^

Protein intake IV (mol C mol C^−1^ day^−1^) was calculated using average individual apparent feed intake rates (g day^−1^) and consumer mass (mol C) measurements as follows:


$$ \mathrm{IV}=\frac{\left\lfloor \frac{\mathrm{Feed}\ \mathrm{intake}\ast \mathrm{Proportion}\ \mathrm{of}\ \mathrm{protein}\ \mathrm{N}\ \mathrm{in}\ \mathrm{feed}}{\mathrm{Molar}\ \mathrm{mass}\ \mathrm{of}\ \mathrm{nitrogen}}\right\rfloor \ast 3.87}{\mathrm{Consumer}\ \mathrm{mass}\ } $$

Lipid intake IH (mol C mol C^−1^ day^−1^) was calculated as


$$ \mathrm{IH}=\frac{\left\lfloor \frac{\mathrm{Feed}\ \mathrm{intake}\ast \mathrm{Proportion}\ \mathrm{of}\ \mathrm{total}\ \mathrm{lipid}\ \mathrm{C}\ \mathrm{in}\ \mathrm{feed}}{\mathrm{Molar}\ \mathrm{mass}\ \mathrm{of}\ \mathrm{carbon}}\right\rfloor }{\mathrm{Consumer}\ \mathrm{mass}\ } $$

We assumed the model default protein C:N ratio (3.7) (Anderson *et al.,* 2020) and obtained SDA value from a study on spiny lobsters (Wang *et al.,* 2021b). We then tuned the other parameters to calibrate the model to data.

### Model calibration to data

For each of the three experimental datasets, we calibrated the model to estimate missing parameters that minimized the difference between predicted and observed intakes and growth rates. For Experiments 1 and 2, this involved estimating:

- fV*, a* parameter that quantifies the relative usage proteins (1-fV for lipids or carbohydrates) to meet energetic costs.
- k^*^_N_ (net protein synthesis efficiency), a parameter that is the conversion efficiency for protein synthesis for growth and replacement biomass.
- Φ Penalty function, a parameter that relates to energetic costs incurred when protein is used to meet demand for absorbed carbon, which only occurs when fV > 0.

**Figure 3:**
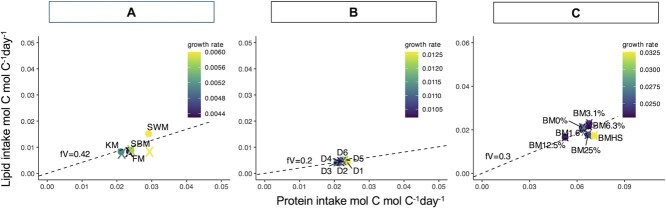
Modelled protein and lipid intakes (coloured X) for the fraction of protein used for metabolic processes (fV) to meet the observed (circles) growth rate associated with each feed. Observed lipid intake, protein intake and growth rates are from Wirtz *et al.,* (2022a, 2022b) (A), Landman *et al.* (2021a) (B) and Landman *et al.* (2021b) (C). The dashed line (slope) indicates average fV across all feeds for the study used to run the model in reverse mode prior to feed-level parameterization. KM, krill meal; SBM, soybean meal; SWM, squid by-product meal. D1–D6: feeds with increasing inclusion levels of phosphatidylcholine. BM0%–BM25%: feeds with increasing inclusion levels of fresh feed (blue mussel). BMHS, blue mussel half-shell feed.

For Experiment 3, two further parameters (absorption efficiency of protein and lipid, β_V_ and β_H_) needed to be estimated as, unlike Experiments 1 and 2, these were not directly measured and provided with the experimental datasets. We then compared model outputs to experimental datasets (Fig. 3).

### Faecal nitrogenous (N) waste output estimation

To examine the effect of accounting for nutrient and macromolecule demand on N waste estimates, we compared outputs from the calibrated GS model (run in forward mode) to a conventional simple bioenergetics approach that does not include stoichiometry (Baldan *et al.,* 2019). The conventional approach assumes a fixed ratio of C:N in feed and consumer and calculates the N waste output from observed crude protein intake (I_V_) as


$$ \mathrm{N}\ \mathrm{waste}\!\!\ \left(\mathrm{mol}\ \mathrm{N}\ \mathrm{mol}\ {\mathrm{C}}^{-1}\ {\mathrm{day}}^{-1}\right)\!=\!\frac{\Big(\mathrm{N}\ \mathrm{intake}\!\times\! \left(1\!-\!{\mathrm{AD}}_{\mathrm{N}}\right)}{3.87} $$

with nitrogen calculated as *N* = crude protein/6.25 (Carter *et al.,* 2025), AD_N_ representing nitrogen digestibility and the value 3.87 representing the protein C:N ratio*.*

To validate the model outputs with experimental data, we required experimental measurements of waste output, which were available only for one study (Experiment 1).

## Results

### Model calibration to experimental observations

The GS model predicted protein and lipid intakes required to meet carbon and nitrogen demands across different growth rates and feed combinations that were constrained by observations. Despite all experimental feeds resulting in similar growth rates within each of the experiments, the model was able to predict differences in protein and lipid intakes for different feeds (Fig. 3). Model predictions were able to match experimental observations for all experimental feeds except squid by-product meal (SWM) (Fig. 3A, yellow X). Protein and lipid intakes for feeds D1–D6 (Fig. 3B) show minimal spread, consistent with the experimental data. Model predictions for different inclusion levels of fresh ingredient (blue mussel, Fig. 3C) also captured the spread of experimental data, with the highest protein intakes predicted for the fresh ingredient feed blue mussel half-shell (BMHS).

Estimated fV values were low for all feeds, ranging from 0 to 0.17 (Table S3). This indicates that, in general, a small proportion of protein intake was used to meet energetic demands. In Experiment 1, fV ranged from 0 to 0.17 with fV > 0 observed for only the soybean meal (fV = 0.17) feed. In Experiment 2, which investigated the influence of phosphatidylcholine supplementation, fV values decreased with increasing inclusion levels, except for feed D4. In Experiment 3, most feeds had 0 fV except for the feeds with 25% inclusion of blue mussels (BM25%; fV = 0.07) and blue mussel half-shell feed (BMHS; fV = 0.13). Protein synthesis efficiency (k^*^_N_) was generally high (>0.75) for all feeds except for squid by-product meal (SWM), which had a lower value of 0.68. The penalty for excess protein (Φ), which is only applicable when fV > 0, was estimated to vary between 0 and 0.69 for all feeds (Table S3).

**Figure 4:**
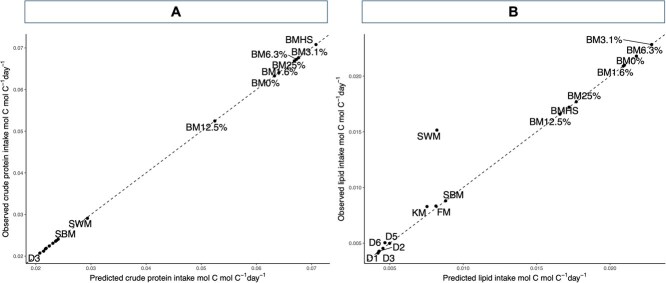
Predicted and observed values of (A) CP intakes (r^2^ = 0.99) and (B) lipid intakes (r^2^ = 0.95) for Experiments 1, 2 and 3. Dashed line indicates 1:1 relationship. KM, krill meal; SBM, soybean meal; SWM, squid by-product meal. D1–D6: feeds with increasing inclusion levels of phosphatidylcholine. BM0%–BM25%: feeds with increasing inclusion levels of fresh feed (blue mussel). BMHS, blue mussel half-shell feed.

Overall, across all three experiments, most of the predicted intakes for lipid and carbohydrates closely aligned with observed values, falling along the 1:1 line with observations (Fig. 4). However, the model underestimated lipid intake associated with squid by-product meal (SWM).

### Comparison of faecal nitrogenous waste estimates

**Figure 5:**
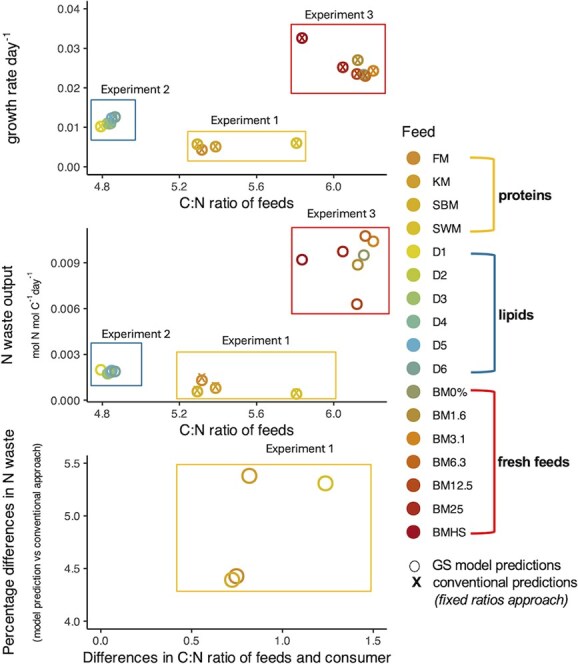
(A) Relationship between feed C:N ratio and growth. (B) Relationship between feed C:N ratio and faecal N waste output. (C) Differences in N waste GS model predictions relative to applying fixed ratios to convert CP intake directly to N waste, in the absence of any consumer demand for protein and lipids. This was done for Experiment 1 only due to data unavailability for Experiments 2 and 3. Feed types by experiment—Experiment 1 (protein sources): KM, krill meal; SBM, soybean meal; SWM, squid by-product meal. Experiment 2 (phosphatidylcholine inclusion): D1–D6, representing feeds with increasing inclusion levels of phosphatidylcholine. Experiment 3 (fresh feed inclusion): BM0%–BM25%: feeds with increasing inclusion levels of fresh feed (blue mussel). BMHS, blue mussel half-shell feed.

Using the calibrated model, we estimated N waste across all feeds (Fig. 5). Among the three experiments, Experiment 3, which tested different inclusion levels of fresh feeds, produced the highest N waste estimates (0.006–0.011 mol N mol C^−1^ day^−1^) and had the highest C:N ratios. On average, N waste in Experiment 3 was 91.7% higher than Experiment 1. Experiment 1, which evaluated different protein sources across feeds, produced the lowest N waste estimates and showed greater variation in N waste (0.0004–0.0013 mol N mol C^−1^ day^−1^) and C:N ratios. Experiment 1 estimated 58.9% less waste compared to Experiment 2. Experiment 2, which tested different lipid inclusion levels, resulted in moderate N waste estimates with little variation among the different feed treatments (0.0017–0.002 mol N mol C^−1^ day^−1^). On average, N waste estimates of Experiment 2 were 79.8% less than Experiment 3.

Calculation of N waste using the conventional approach, which does not account for the C:N ratio of the consumer, was only possible for Experiment 1 as this required apparent protein digestibility data. Both methods estimated the highest N waste output for fish meal (FM) and the lowest for squid by-product meal (SWM) (Fig. 5B). On average, the model predicted 4.9% more waste than the conventional approach. The magnitude of differences between the two N waste methods did not appear to be driven by differences in the C:N ratios of feeds versus the consumer.

## Discussion

Using a novel modelling approach based on organismal homeostasis (GS), we show for the first time that the GS framework can be applied to data-poor and emerging aquaculture species. Through a case study on *T. australiensis*, we investigated the influence of aquafeed ingredient type, quality and quantity on growth and waste output and compared model outputs to opportunistically available experimental datasets. Our study reveals the following: (i) GS predicts higher waste associated with feeds, including a fresh ingredient component. (ii) With limited data, the model could predict protein and lipid intakes and N waste across a range of feed ingredients. Whether or not these findings are generally applicable to aquaculture would require substantially more data to validate waste outputs for this species. However, with directed experimental work, this could be achieved.

We used opportunistically available data for our initial model exploration and note that the experiments were not designed as GS experiments, but rather to investigate the influence of different feed ingredient types and sources on the growth performance of *T. australiensis* from a feed formulation and nutrition perspective. While there is a paucity of data relating to macronutrient requirements for *T. australiensis* growth, we do know that lobsters require higher quantities of dietary CP as they grow, unlike finfish with decreasing protein requirements (Spanier and Lavalli, 2013). Experiment 1 aimed to identify suitable protein sources for *T. australiensis* feed formulation. The high intake of squid by-product meal (SWM) (Fig. 4A, yellow dot) can potentially be attributed to overestimation of apparent feed intake as squid by-product meal produced an unstable feed pellet (high dry matter loss in water) compared to other protein sources tested (Wirtz *et al.,* 2022b). The overestimation of apparent feed intake for squid by-product meal (SWM) in the experiment (Wirtz *et al.,* 2022b) may have also affected our estimates of protein synthesis (k^*^_N_) values, which were lowest for squid by-product meal (0.68). In reality, protein synthesis is a complex process influenced by different feed ingredients, their protein and energy content, metabolic processes (Wang *et al.,* 2021a, 2021b), life stages and the surrounding environment (Small *et al.*, 2015). SDA, which quantifies energetic costs associated with feed intake, absorption and assimilation can also affect protein synthesis. There is evidence that lower values of SDA can increase protein synthesis efficiency (Mente *et al.,* 2002; Wang *et al.,* 2021a, 2021b; Carter *et al.,* 2025). However, we did not have data on feed and species-specific SDA for the lobsters in this study.

One of the key features of the GS model is the inclusion of a metabolic penalty function when protein is used to meet energetic costs (i.e. when fV > 0). We anticipated the penalty function Φ to be high given the evidence that crustaceans preferentially use amino acids as an energy substrate (Li *et al.,* 2021; Williamson *et al.,* 2023). Estimated Φ values ranged between 0 and 0.69 for all feeds with the highest penalty predicted for the feed D4 in Experiment 2 (Table S3). This implies a heavy energetic cost will be incurred when protein is provided in excess according to the GS modelling framework. Since we assumed model defaults for protein absorption efficiency for this feed (β_V_ = 0.69), the high energetic cost may have arisen due to a potential underestimation of protein absorption efficiency, β_V_. This highlights the clear need for targeted experiments with complete measurements of absorption and assimilation efficiencies for all macronutrients. The penalty function provides a way to ascertain an organism’s metabolic costs of dealing with excess nutrients, which is useful to identify threshold elemental ratios and stoichiometric knife-edges of nutrients (Anderson *et al.,* 2020) in feeds to design optimal feeds for aquaculture. In the absence of the penalty function, there are no adverse impacts of excess nutrients on an organism’s growth, and they can feed on various diets to maximize limiting nutrients (Raubenheimer and Simpson, 2018; Anderson *et al.,* 2020). The regulation of excess nutrients involves complex metabolic processes (Raubenheimer and Simpson, 2018), which vary among different species (Raubenheimer and Simpson, 2003; Anderson *et al.,* 2020) and developmental stages (Al Shareefi and Cotter, 2019; Anderson *et al.,* 2020).

Lipids are an important energy source in well-fed lobsters (Rodríguez-Viera *et al.,* 2017), and some studies indicate that lobsters preferentially utilize proteins and lipids as energy sources (Perera *et al.,* 2005; Rodríguez-Viera *et al.,* 2017) and that generally carbohydrates are well-digested but poorly utilized (Rodríguez-Viera *et al.,* 2014, 2022; Munian *et al.,* 2021). Due to this, data on lipid digestibility are more readily available than for carbohydrates, and hence, lipids are the current focus of the study. In general, lobsters require lower dietary lipid levels compared to finfish; however, cholesterol supplementation is crucial as it is an essential dietary resource (Wigglesworth and Griffith, 1994; Turchini *et al.,* 2022; Landman *et al.,* 2024). Experiment 2 investigated the role of phosphatidylcholine, an important dietary lipid to identify ‘optimal’ levels of its inclusion where growth performance is maximized. Since all experimental feeds contained the same amount of CP, the slight differences in growth performance may be attributed to changes in lipid type and inclusion levels. While carbohydrate requirements are commonly examined as non-protein nutrients, there is limited information on the carbohydrate requirements for lobsters.

There is limited experimental knowledge on the relative usage of proteins versus non-nitrogenous macromolecules for meeting energetic costs (as defined by fV) of lobsters (Wang *et al.,* 2021b; Williamson *et al.,* 2023). The low fV values estimated for all feeds indicate that most of the protein intake was allocated to growth, and energetic costs were met by either lipids or carbohydrates (Fig. 3, Table S3). In Experiment 1, fV for soybean meal (SBM) was estimated to be 0.17, implying that some of the ingested protein is being used to meet energetic costs. In Experiment 2, which investigated the influence of phosphatidylcholine supplementation, fV decreased with increasing inclusion levels, with the exception of feed D4 suggesting that the supplementation may promote better utilization of proteins and lipids for their intended purposes. In Experiment 3, the high penalty (0.51) and fV (0.13) associated with fresh feed blue mussel half-shell (BMHS) suggests a high energetic cost, which is incurred when protein is consumed in excess to meet energetic costs. However, this cost is offset by the highest growth rate observed in lobsters fed the fresh blue mussel half-shell (BMHS) feed, highlighting the complex nature of feed selection, intake and assimilation. Further empirical data on the relative use of protein for energy in this species are needed to cross-validate our estimates based on GS.

Our study shows that fresh feeds are associated with higher protein intake, growth rates and high N waste output, which agrees with current literature (Landman *et al.*, 2021b). Fresh ingredients in feeds, especially mussels, are known to promote superior growth performance in lobsters compared to formulated feeds (Landman *et al.*, 2021b). The causal mechanisms are largely unresolved, although several hypotheses suggest that fresh ingredients may be highly digestible and possess desirable physical and nutritional characteristics (Perera and Simon, 2015; Landman *et al.*, 2021b). However, it is well established that using fresh feeds increases waste production and degradation of water quality (Nankervis and Jones, 2022), thereby necessitating development and use of nutritionally balanced formulated feeds. In the GS model, higher metabolic penalties were also estimated for the feeds containing fresh blue mussel, suggesting protein was in excess. N waste estimates for Experiment 2 were relatively similar across all feeds, as expected due to the similarity in their formulations. N waste estimates varied the most in Experiment 1, which investigated the influence of different protein sources. Squid by-product meal (SWM) feed was associated with lowest N waste, likely due to its high intake, absorption and assimilation (Wirtz *et al.,* 2022b). In contrast, FM feed had the highest N waste output, due to lower absorption efficiency compared to the other three feeds. Given the rising cost of protein sources and ongoing concerns about water quality in lobster aquaculture, further research is needed to optimize formulations that balance performance with environmental sustainability (Spanier and Lavalli, 2013).

## Conclusion

Our study used growth and waste output as a quantitative measure of how feeds fare because of data limitation, but the model predicts other metabolic metrics like C and N budgets, elemental gross growth efficiency, respiration and excretion, which would be highly useful for aquaculture. We highlight the need for a new generation of theoretical and experimental studies with measurements of food selection, consumer biomass, consumer body composition, metabolism (including estimates of metabolic penalties that consider protein turnover) and associated physiology to validate the GS model for aquaculture. While aquaculture’s main goal is to maximize growth performance and minimize waste output, NG emphasizes that food is consumed to maximize overall fitness, which depends on different life stages and includes various factors like survival, ease of food availability, growth, fecundity and reproduction (Simpson and Raubenheimer, 1995; Raubenheimer *et al.,* 2009; Raubenheimer and Simpson, 2018; Anderson *et al.,* 2020).

Future exploration and data are needed to evaluate GS framework’s potential as a predictive tool to better understand how different macromolecule combinations and nutrient stoichiometries impact growth performance of aquaculture species at different life stages and how feeds with different nutrient stoichiometries fare in the environment. Subsequent incorporation of the GS framework into ecosystem and biogeochemical models could yield important insights into how elemental cycling operates through marine ecosystems and help further develop tools to support an EAA.

## Ethics declarations

This study did not require any ethics approval as it used published datasets on slipper lobster nutrition.

## Supplementary Material

Web_Material_coaf066

## Data Availability

The code and datasets generated and/or analysed during the study are available in the GitHub repository—https://github.com/Sustainable-Aquafeeds-Project/GS_lobstermodel
