# Supplementary material for: Geometric stoichiometry model yields relevant insights for assessing nutrient-related environmental impacts of aquaculture
Source: Conserv Physiol. 2025 Sep 15;13(1):coaf066. doi: 10.1093/conphys/coaf066 (PMC12448620; doi:10.1093/conphys/coaf066)
Supplement: Web_Material_coaf066 [file web_material_coaf066.pdf]

Supplementary material

Geometric stoichiometry model yields relevant insights for assessing nutrient-related environmental impacts of aquaculture

Table 1: Geometric Stoichiometry model equations from Anderson et al. 2020.

**Equations**	**Units**	**Description**
**Reverse mode**		
1. $D_N = \frac{\tau+G}{\kappa^*{}_N}$	mol N mol N^{-1} d^{-1}	Demand for dietary protein where tau and G are biomass turnover and growth rate, respectively. k^*_N is the maximum nitrogen synthesis efficiency
2. $D_V = \frac{\theta_V}{\theta_Z} D_N$	mol C mol C^{-1} d^{-1}	Corresponding demand for absorbed protein
3. $D_X = \frac{(\theta_Z-\theta_V)(\tau+G)}{\theta_Z}$	mol C mol C^{-1} d^{-1}	Extra demand for C given that the C:N ratio of consumer biomass is greater than that of protein
4. $DA = DX + \xi + \eta(IV + IH)$	mol C mol C^{-1} d^{-1}	Demand for absorbed C is the sum of DX, other basal costs, and specific dynamic action
5. $\Omega = fV\phi DA + (fV\phi)^2 DA + (fV\phi)^3 DA + ..$ $= DA\sum_{I=1}^{\infty} (fV\phi)^I = D_A(\frac{1}{1-\phi fv} - 1)$	mol C mol C^{-1} d^{-1}	Total penalty cost: Sum of recursive infinite series as the penalty compounds on itself, depending on whether proteins or carbohydrates are used
6. $\beta VIV = DV + fVDA + fV\Omega$	mol C mol C^{-1} d^{-1}	Allocation of absorbed protein
7. $\beta HIH = (1 - fV)DA + (1 - fV)\Omega$	mol C mol C^{-1} d^{-1}	Allocation of absorbed carbohydrate
8. $IH = \frac{\frac{Dv+a(D_X+\xi)}{\beta_V-\eta a}+\frac{D_X+\xi}{\eta}}{\frac{\beta_H(1-fv\phi)}{\eta(1-fv)}-\frac{\eta a}{\beta_V-\eta a}-1}, a = \frac{fv}{(1-\phi fv)}, fv < 1$	mol C mol C^{-1} d^{-1}	intake of carbohydrate, IH
9. $I_V = \frac{\beta_H I_H(1-\phi fv)}{\eta(1-fv)} - \frac{Dx+\xi}{\eta} - I_H, fv < 1$	mol C mol C^{-1} d^{-1}	intake of protein, I_V
10. $I_V = \frac{D_V+\frac{D_X+\xi}{1-\phi}}{\beta_V-\frac{\eta}{1-\phi}}, fv = 1$	mol C mol C^{-1} d^{-1}	intake of protein, I_V
11. $R = \tau + (1-\kappa_N^*)D_V + \xi + \eta(I_V + I_H) + \Omega$	mol C mol C^{-1} d^{-1}	respiration budget
12. $E = \frac{\tau}{\theta_Z} + \frac{(1-\kappa_N^*)D_V}{\theta_V} + \frac{fv}{\theta_V}\left(D_X + \xi + \eta(I_V + I_H)\right) + \frac{fv}{\theta_V}\Omega$	mol N mol C^{-1} d^{-1}	excretion budget
13. $W_C = (1-\beta_V)I_V + (1-\beta_H)I_H$	mol C mol C^{-1} d^{-1}	faecal carbon
14. $W_N = (1-\beta_V)\frac{I_V}{\theta_V}$	mol N mol C^{-1} d^{-1}	faecal nitrogen
**Forward mode**		
1. $I_{Vm} = \frac{\theta_V \tau}{\beta_V k_N^* \theta_Z}$	mol C mol C^{-1} d^{-1}	The protein intake needed to meet the maintenance cost of biomass turnover, I_{Vm} (mmol C mmol C^{-1} d^{-1}), is (as in Eqs 1, 2 of the reverse models, but with G = 0)
2. $D_{Xm} = \frac{(\theta_Z-\theta_V)\tau}{\theta_Z}$	mol C mol C^{-1} d^{-1}	Additional C needed to make up the demand for C for the replacement biomass, D_{Xm}, is

3. $I_{Hm}^{*} = \frac{\eta I_{Vm}+D_{Xm}+\xi}{\beta_H-\eta}$	mol C mol C^{-1} d^{-1}	Carbohydrate intake accounting for losses to absorption and specific dynamic action when D_{Xm} is met solely using carbohydrates
4. $I_{VG}^{n} = \frac{\theta_V}{\beta_V k_N^{*} \theta_Z}$	mol C mol C^{-1} d^{-1}	The optimal intake requirement for protein for growth (in the absence of maintenance)
5. $D_{XG}^{n} = \frac{(\theta_Z-\theta_V)}{\theta_Z}$	mol C mol N^{-1}	The corresponding equation for demand for additional C
6. $I_{HG}^{n*} = \frac{\eta I_{VG}^{n}+D_{XG}^{n}}{\beta_H-\eta}$	mol C mol C^{-1} d^{-1}	The corresponding equation for intake of carbohydrate
7. $\theta_{HVG}^{*} = \frac{I_{HG}^{n*}}{I_{VG}^{n}}$	mol C mol N^{-1}	The C:N ratio of growth
8. $\theta_{HV}^{*} = \frac{I_{Hm}^{*}+(I_V-I_{Vm})\theta_{HVG}^{*}}{I_V}$	mol C mol N^{-1}	For a given protein intake I_V, the carbohydrate to protein ratio that gives optimal nutrition, θ_{HV}^{*}
9. $I_H^{*} = I_V \theta_{HV}^{*}$	mol C mol C^{-1} d^{-1}	The maximum utilisable carbohydrate corresponding to a given protein intake, I_H^{*}
10. $I_{HU} = min\,[I_H, I_H^{*}]$	mol C mol C^{-1} d^{-1}	If I_H > I_H^{*}, then carbon is in stoichiometric excess. The carbohydrate that is allocated to growth and metabolism is thus the minimum of I_H and I_H^{*}
11. $C_X = (\beta_H - \eta)(I_H - I_{HU})$	n.a	The excess rate of C intake, C_X
12. $f_V = \frac{-a+bd-bc}{-a+\phi bd-d-bc}, a = \beta_V I_V,$ $b = \frac{\theta_V}{k_N^{*}(\theta_Z-\theta_V)},$ $c = \xi + \eta(I_V + I_{HU}), d = \beta_H I_{HU}$	n.a	f_V , model parameter which quantifies relative usage of proteins and carbohydrates for energy is calculated by rearrangement of I_H and I_V in Eqs. 6 and 7
13. $D_{CX} = \frac{\beta_H I_{HU}(1-\phi f_V)}{(1-f_V)} - \left(\xi + \eta(I_V + I_{HU})\right), f_V < 1$	mol C mol C^{-1} d^{-1}	Further rearrangement of these equations gives D_{CX}, allowing calculation of G
14a. $G = \frac{\theta_Z D_{CX}}{(\theta_Z-\theta_V)} - \tau, f_V < 1$	d^{-1}	Growth when fV is less than 1, i.e., energetic costs are met by carbohydrates
14b. $G = \frac{\beta_V I_V - \frac{\xi+\eta(I_V+I_H)}{1-\phi}}{\left(\frac{\theta_V}{\kappa_N^{*}\theta_Z}+\frac{\theta_Z-\theta_V}{(1-\phi)\theta_Z}\right)} - \tau, f_V = 1$	d^{-1}	Growth when fV is equal to 1, i.e., energetic costs are met solely by proteins
15. $R = \tau + (1 - k_N^{*})D_V + \xi + \eta(I_V + I_H) + \Omega + C_X$	mol C mol C^{-1} d^{-1}	respiration budget
16. $E = \frac{\tau}{\theta_Z} + \frac{(1-k_N^{*})D_V}{\theta_V} + \frac{f_V}{\theta_V}\left(D_X + \xi + \eta(I_V + I_H)\right) + \frac{f_V}{\theta_V}\Omega$	mol N mol C^{-1} d^{-1}	excretion budget
17. $W_C = (1 - \beta_V)I_V + (1 - \beta_H)I_H$	mol C mol C^{-1} d^{-1}	faecal carbon
18. $W_N = (1 - \beta_V)\frac{I_V}{\theta_V}$	mol N mol C^{-1} d^{-1}	faecal nitrogen

Supplementary material

Geometric stoichiometry model yields relevant insights for assessing nutrient-related environmental impacts of aquaculture

Table 2 :Model parameters and definitions (tuned parameters highlighted) from Anderson et al 2020.

Parameter	Definition	Value	Unit of measure	References
β_V	Absorption efficiency: protein	Varied across feeds	Dimensionless	Landman et al. 2021a<br>Landman et al. 2021b<br>Wirtz et al., 2022a
β_H	Absorption efficiency: lipids	Varied across feeds	Dimensionless	Landman et al. 2021a<br>Landman et al. 2021b<br>Wirtz et al., 2022a
k^*_N	Maximum net synthesis efficiency: N	tuned	Dimensionless	
l (equivalent to growth rate)	Biomass turnover	tuned	d^{-1}	
ξ	Other basal costs	tuned	d^{-1}	
η	Specific dynamic action	0.0259	Dimensionless	Wang et al 2021b
θ_V	Protein C:N ratio	3.7	mol C mol N^{-1}	Model default
θ_Z	Consumer C:N ratio	4.57	mol C mol N^{-1}	Codabaccus et al. 2020; Wirtz et al. 2022b
f_V	Fraction of Dc met using protein	0-1,tuned	d−1	
φ	Penalty function	$0 \le \varphi \le 1$, tuned	d^{-1}	

Supplementary material

Geometric stoichiometry model yields relevant insights for assessing nutrient-related environmental impacts of aquaculture

Table 3: : Estimated parameters for experiments 1-3. Experiment 1 feeds: FM: fish meal, KM: krill meal, SBM: soybean meal, SWM: squid by-product meal; Experiment 2 feeds: D1-D6: feeds with successive inclusion levels of phosphatidylcholine (a phospholipid); Experiment 3 feeds: BM0%- BM25% feeds with different inclusion levels of fresh blue mussel in formulated feeds, BMHS= blue mussel half shell. Parameters: fV is the fraction of protein used to meet energetic costs; $\boldsymbol{k^*_N}$ *net nitrogen synthesis efficiency (protein synthesis efficiency); Φ penalty to incur additional costs when protein is in excess.*

Experiment	Experimental feed	fV	k^*_N	Φ	β_V	β_H
1	FM	0	0.94	0	-	-
1	KM	0	0.99	0	-	-
1	SBM	0.17	0.89	0.047	-	-
1	SWM	0	0.68	0	-	-
2	D1	0.11	0.90	0.043	-	-
2	D2	0.078	0.87	0.352	-	-
2	D3	0.076	0.923	0	-	-
2	D4	0.083	0.837	0.687	-	-
2	D5	0.028	0.885	0.499	-	-
2	D6	0	0.836	0	-	-
3	BM0%	0	0.814	0.52	0.444	0.361
3	BM1.6%	0	0.828	0.53	0.488	0.397
3	BM3.1%	0	0.813	0.5	0.431	0.347
3	BM6.3%	0	0.825	0.54	0.409	0.35
3	BM12.5%	0	0.79	0.53	0.556	0.435
3	BM25%	0.07	0.807	0.508	0.462	0.433
3	BMHS	0.13	0.859	0.507	0.52	0.51
